# Physicians’ ethical responsibilities in relation to the climate and further environmental crises: a review of academic publications

**DOI:** 10.1007/s11019-026-10339-1

**Published:** 2026-03-09

**Authors:** Cristian Timmermann, Katharina J. Pascale Wabnitz, Kerstin Schlögl-Flierl, Verina Wild

**Affiliations:** 1 Institute for Ethics and History of Health in Society, Faculty of Medicine, University of Augsburg, Augsburg, Germany; 2 Moral Theology, Faculty of Catholic Theology, University of Augsburg, Augsburg, Germany

**Keywords:** Responsibilities of healthcare professionals, Climate change, Medical duties, Pollution, Ethical obligations to the environment

## Abstract

The climate and further environmental crises have motivated calls for the medical profession to act by taking on additional responsibilities. These calls to assume responsibilities towards environmental protection and to systematically consider the health impacts of these crises greatly vary in their scope and demandingness. Through a review of journal publications, we have mapped the various calls for physicians to take on responsibilities in relation to these crises as individuals and as a professional group. These professional responsibilities, obligations or duties were grouped in four broad categories of physicians’ roles as (i) medical practitioners, (ii) medical scientists, (iii) facility (co-)managers, and (iv) citizens. In sum, these responsibilities go beyond actions within the individual patient-physician relationship and setting, demanding from physicians to get involved within their institution, their community, engage with policy-makers, and also concern themselves with the health effects of environmental changes also on distant others, such as people in other parts of the world and future generations.

## Introduction

Environmental and climatic changes are threatening health (van Daalen et al. 2024). It has been repeatedly stated over the last three and a half decades that physicians should make the health consequences of environmental and climatic deterioration their concern (Leaf 1989; Crowley 2016). Recent evidence from a global survey confirms that physicians are aware of the health impacts of climate change and mostly willing to act, including as advocates in the public or political realms. However, one identified barrier in this study was that “public engagement [could be] too risky for them professionally or personally” (Kotcher et al. 2021).

The recently updated version of the International Code of Medical Ethics (ICoME) by the World Medical Association (2022), explicitly mentions that “The physician also has a responsibility to contribute to the health and well-being of the populations the physician serves and society as a whole, including future generations” and that “The physician should strive to practise medicine in ways that are environmentally sustainable with a view to minimising environmental health risks to current and future generations” (Parsa-Parsi 2022). However, what exactly this expanded responsibility entails and how to balance duties towards the individual patient with duties towards society and future generations, is not yet clear.

Hence, there is uncertainty among physicians regarding what the environmental and climatic crises mean for their profession and how they should respond (Boland and Temte 2019; Tarver and Macpherson 2025; Yassaie and Brooks 2025). At the same time, physicians are being asked to simultaneously respond to multiple other crises, such as defending the availability of healthcare services from drastic budget cuts, handling the opioid epidemic and securing reproductive health services (Chimonas et al. 2021). Moreover, prevailing health burdens need to be addressed while they are being aggravated through pollution and climate change (Faergeman 2007).

Therefore, our aim in this review is to summarize existing academic literature by reviewing, mapping out, and discussing what responsibilities in relation to the environment are ascribed to practicing physicians by their peers, bioethicists and other health researchers. Our motivation to carry out this review is that physicians currently face a large number of varied calls to assume environment-related responsibilities without having a clear overview of which calls are being issued, which demands for action have broad support in the literature and which calls have not been widely raised. We therefore focus on appeals made in the present and not on tasks assigned primarily to a future generation of physicians. The review thereby lays the groundwork for further normative analysis on whether the identified aspects (or others) *should* constitute part of physicians’ responsibilities, how these responsibilities are grounded, which tensions might arise and how these can be addressed, and how this might affect professional identity formation in medical education.

## Methodology

Our research question was: Which ethical responsibilities in relation to the climate and further environmental crises do physicians assume or are being ascribed?

To answer this question, we opted for a non-systematic review of academic publications in line with general principles of a narrative review (Sukhera 2022). The reason for following this approach is that authors with different professional backgrounds often use normative and professionalism-related concepts differently and even within the same discipline we can observe differing understandings of concepts. For instance, calls to take on responsibilities were sometimes framed very explicitly as ethical or moral. Such calls were framed e.g. as “imperative for health care practitioners”, “responsible for”, or “moral duty” while other authors preferred more general terms, such as “should”, “must” and “do their share”. As there is no consistency in describing and framing ethical responsibilities by different stakeholders, a non-systematic review gave us the necessary freedom to synthetise very diverse calls for action and to map the key ideas on physicians’ responsibilities in relation to the climate and further environmental crises.

Our search aimed to identify a large diversity of published calls from, for, and towards physicians without aiming to be all-encompassing. We included articles of all types, including opinion pieces and letters, and did not set any time or geographic boundaries. Between January and May 2024, we carried out extensive searches on Web of Science, PubMed and GoogleScholar, using key words such as “physicians ought”, “doctors ought”, “physicians should”, “doctors should”, “physicians have to”, “physicians must”, “doctors have to” in combination with “environmen*”, “climate”, “nature” or “sustainab*”, and “health”, iteratively adapting search terms to find relevant publications. The databases were chosen to allow us to identify calls in and outside the health sciences. We snowballed further publications using unsystematic forward and backward citation screening of potentially relevant references. Further salient publications that were released after the search period were also included.

Our inclusion criteria were: Sources had to (i) be in English, German, or Spanish, (ii) deal primarily with responsibilities of physicians in relation to the climate and environmental crises, and (iii) contain clearly identifiable ethical demands on physicians’ activities in relation to the climate and environmental crises.

In terms of exclusion criteria, we did not include related articles that turned up in our search but focussed on (i) indoor air pollution, (ii) military contexts, (iii) tobacco use, (iv) nuclear disasters, (v) awareness of climatic and environmental factors among medical students, (vi) medical education and medical students (except when dealing with continued education), (vii) empirical studies collecting opinions of individual physicians, and (viii) transmission of diseases from animals. The reasons for excluding issues concerning medical education was, firstly, to narrow down the quantity of papers and, secondly, to adhere to our intention to focus on responsibilities that already practising physicians are presently being asked to assume. We also excluded empirical studies on surveying attitudes towards assuming environmental responsibilities, as they did not invoke broader calls for action. Studies relating to transmission of animal diseases were excluded, as the richness of the subject, ranging from pandemic prevention to animal welfare, and its ethical complexities merits a separate analysis.

As we were interested in including statements from a comprehensive and diverse range of sources, we had rather broad quality control criteria: (i) book chapters needed to come from international publishers, and, (ii) regarding sources identified via GoogleScholar, publications had to be additionally indexed in Web of Science, PubMed, Scopus or Scielo, or backed by medical associations.

To report our findings, we mapped articles according to inductively developed categories in a two-step process. In the first step, we identified 14 categories on the basis of sample quotes, which after further analysis and discussion among the authors was reduced by two categories and expanded again by two new categories. In a second step, we grouped these 14 in categories in four inductively developed overarching categories and nine subcategories in relation to the different roles medical doctors play. The disciplinary background of the authors involved in this process is bioethics, environmental ethics, philosophy, medicine, theology and public health. We considered having reached data saturation when further searches did not yield findings of sufficiently new ideas.

## Results

**Fig. 1:**
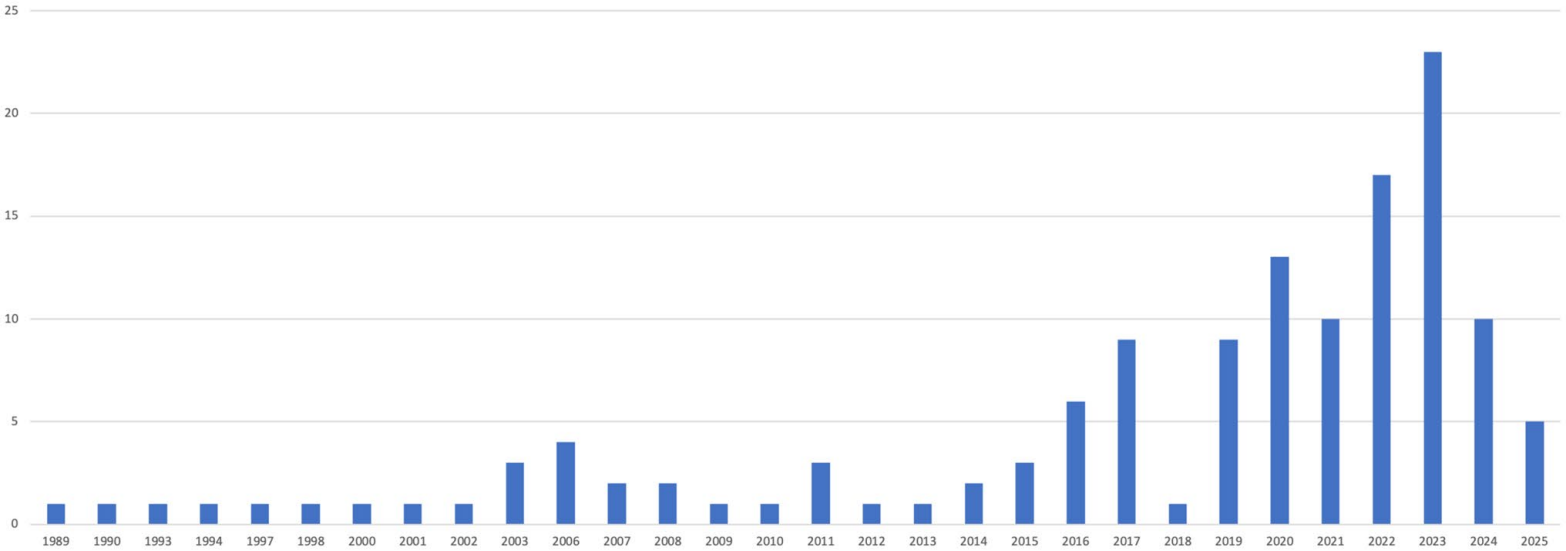
Distribution of publications per year

**Fig. 2:**
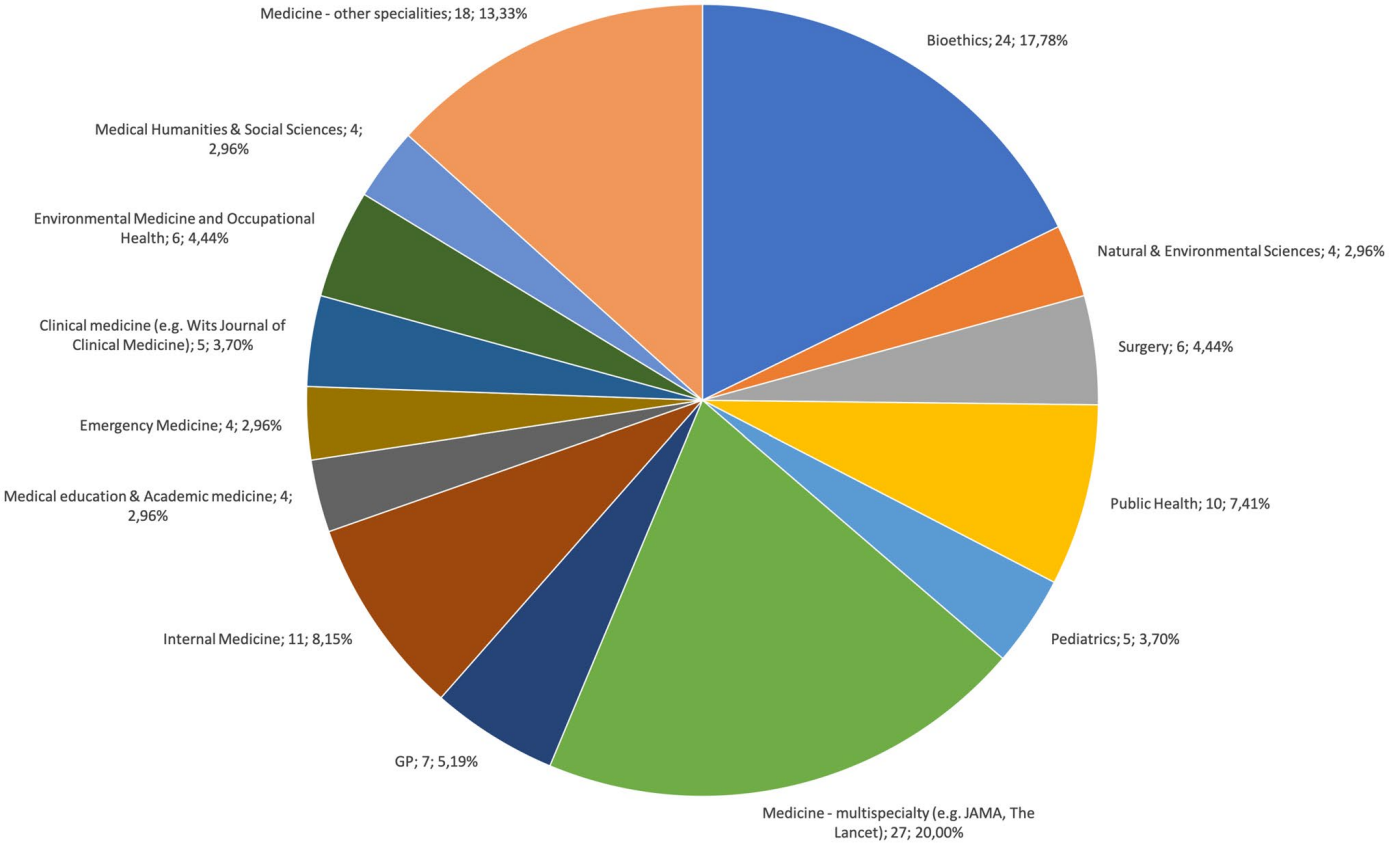
Discipline and medical specialty of sources

We identified a very wide range of responsibilities physicians are asked to assume (see Table 1) published between 1989 and 2025 (see Fig. 1). Calls for assuming ethical responsibilities were usually phrased in terms of “should”, “have the responsibility”, “have the duty” and “must”. Most calls were raised by physicians in medical journals (see Fig. 2). The language distribution of the included calls was English (n = 119), Spanish (n = 6) and German (n = 10). We have clustered these in four categories in relation to diverse professional roles of physicians. These were responsibilities primarily based on their role as (i) medical practitioners, (ii) medical scientists, (iii) facility (co-)managers. We also included a fourth category (iv) responsibilities as citizens, that is irrespective of their professional roles.

**Table 1:** Overview of responsibilities identified in the literature with number of sources addressing each identified responsibility

*Responsibilities based on role as medical practitioners*	
Responsibilities to learn	(n = 22)
Responsibilities to inform patients	(n = 21)
Responsibilities to protect vulnerable groups from environmental harms	(n = 9)
*Responsibilities based on epistemic authority as medical scientists*	
Responsibilities to identify new risks to health arising from the climate and environmental crises	(n = 11)
Responsibilities due to being perceived as role model with medical knowledge	(n = 8)
Responsibilities to publicly warn others of environmental concerns and engage in advocacy	(n = 56)
*Responsibilities based on influence on management*	
Responsibilities to reduce the environmental footprint of the healthcare sector	(n = 42)
Responsibilities to prepare the healthcare sector for environmental and climatic disruptions	(n = 16)
*Responsibilities as citizens*	
Responsibilities as members of society without appeal to special position, skills or knowledge	(n = 7)

### Responsibilities based on role as medical practitioners

A large proportion of the responsibilities referred to the physician’s role as a medical practitioner. Physicians perceived themselves as being in a unique position to inform patients and clarify questions in their direct encounter during check-ups and treatments. Furthermore, physicians sought to understand the effects of emerging environmental and climate changes for their medical practice. Therefore, much of these responsibilities are knowledge-related: to acquire and pass on information on these emerging health threats and how to respond to them.

#### Responsibilities to learn

Central to many calls for action were appeals to physicians to inform themselves. We found calls to *become knowledgeable* and *familiarize oneself* with how climate change and environmental deterioration affect health risks and healthy living (Faergeman 2007; Leaf 1989; Levine 2016), and more generally, with the health impact of climate change and medical waste on health and the environment (Milovac 2023). In rare cases, these calls used weaker appeals, such as “having to occupy oneself” (in the German original: *sich befassen*) with the health impacts of climate change (Kenner et al. 2023).

The responsibility to learn included anticipating patients’ climate-related healthcare needs for adequate diagnosis and treatment (Sanabria and Duram 2017; Grandi et al. 2023). Knowledge was also judged to be important to be alert about options regarding prevention, and for identifying the necessity for referal to specialty care (Gehle et al. 2011). Furthermore, there were brief references to local contexts by specifying that physicians ought to understand how the specific communities they serve are affected by climate change (Giudice and Rublee 2023) and other environmental factors (Warren, Walker Jr, and Nathan 2002). Moreover, climate change makes it even more important for physicians to stay up to date on emerging infectious diseases in their communities and areas where their patients are likely to travel (Parker et al. 2019). Due to the impact of climate change and pollution on the worsening of allergic symptoms, physicians should learn to understand pollen and air quality forecasts to counsel patients and caregivers (Di Cicco et al. 2020).

In some instances, calls to acquire knowledge demanded to get familiar with the bigger picture, by stating that physicians must zoom beyond public health to view the health of the planet and understand complex natural systems (Kemple 2020). Similarly, physicians should educate themselves on the scientific basis of calls for ecological sustainability, recognize its ethical and technical challenges (Katz 2021) and understand the risks of inaction and benefits of climate action (Huang et al. 2015).

Some physicians working with children and on reproductive health seemed to show special concerns on gaining awareness of environmental risks (Moshammer et al. 2006). For instance, they appealed to their colleagues to become knowledgeable on environmental threats to counsel caregivers to protect children (Di Cicco et al. 2020; Gozderesi et al. 2022) and to recognize the connection between climate change, environmental injustices, and adverse reproductive health outcomes (Martin et al. 2022).

In most occasions the papers – explicitly or implicitly – justified these appeals with the medical practitioner role of physicians, which is to care for patients’ health. In a few occasions, these demands were justified by appealing to reasons outside the classic patient-physician relationship. We identified calls that claimed that physicians should become knowledgeable to satisfy public expectations (Guidotti et al. 1998), and to fulfil their role as trusted communicators (Sarfaty and Abouzaid 2015). It was also requested that physicians need to become knowledgeable about the problems of medical waste and how to dispose it responsibly (McCally and Cassel 1990). Another argument was to state that an understanding of the health impacts of climate change was necessary to educate the next generation of physicians (Moloo et al. 2024).

#### Responsibilities to inform patients

In line with autonomy as an important ethical principle in medical practice, we found calls to specify patient information in view of environmental degradation and climate change.

We could find several calls discussing a directive nature in counselling. These calls focussed on the patients’ need for information on the environmental footprint of the health interventions they are consenting to and the health products they require, e.g. a so-called “green informed consent” (Richie 2023; Cohen et al. 2024). Others pointed out that physicians should counsel patients about the effects of environmental degradation and the environmental and social consequences of their lifestyle (Donohoe 2003), educate their patients on how to reduce greenhouse gas emissions (Parker et al. 2019), and inform about behavioural change in view of climate change (Schwartz et al. 2006; Palmeiro-Silva et al. 2020).

Physicians also suggested to their colleagues to emphasise co-benefits, e.g. by raising patients’ awareness about the climate and health benefits of active transportation and plant-based diets (Parker et al. 2019; Lu 2016; Bhagratie and Iyengar 2021; McCutcheon et al. 2022; Di Cicco et al. 2020), encouraging patients to use less motorized transportation (Pradere et al. 2023) and more generally, to advocate for behaviours and lifestyles that promote planetary health (Iyer et al. 2021; Moser et al. 2017; Wabnitz et al. 2020). One paper argued that recommending plant-based diets is a highly effective tool to promote health and protect the environment, and that failing to use this tool would harm patients and thus constitute an unethical behaviour (Storz 2020).

Some of these calls applied only to specific healthcare contexts. For instance, for physicians working in hospitals, there were calls to inform patients on environmental hazards after being discharged from hospitals to prevent complications (Giudice and Rublee 2023). Occupational health physicians were asked to inform employees on the risks of climate change in their work environments (Grandi et al. 2023). In the case of major events caused by contamination with toxic substances, such as lead, physicians should provide information to their patients about the nature of the risks and strategies to mitigate negative effects on health (Taylor et al. 2017).

Another approach was to ask physicians to advise patients on how they can protect themselves against environmental hazards, for instance, by providing them with information on do-it-yourself air cleaners (Patel et al. 2021). Physicians were also encouraged to inform their patients about heat, pollen, and mould in order to manage symptoms of allergies and asthma (Sanabria and Duram 2017). More broadly, physicians were asked to provide patients with climate-related information on how to protect themselves, their families, and communities (Senay et al. 2021).

#### Responsibilities to protect vulnerable groups from environmental harms

There were two patient groups – children and older adults – whose increased vulnerability led to calls for physicians to assume special responsibilities for these groups. Responsibilities towards children were often framed as a duty. As climate change frequently leads to higher temperatures and humidity, physicians should advice parents on health problems linked to poor ventilation, mould grow and on the health risks of neglecting maintenance of heating installations (Moshammer et al. 2006). Paediatricians were asked to detect and treat effects of environmental chemical or toxic pollution (Morariu et al. 2018). Further, they should talk to children and adolescents about whether climate developments are triggering fear and worries (Fuchsig and Scholl-Bürgi 2022).

Furthermore, the impact of the environment on children’s health included responsibilities to advocate at the community level and push for regulations for a healthy environment for children (Moshammer et al. 2006; Gozderesi et al. 2022; Di Cicco et al. 2020). Moreover, there were calls to protect planetary health entrusted to humankind for the sake of all children (Marais et al. 2021; Williams et al. 2023; Donohoe 2003).

Appeals to pay special attention to health hazards for older adults were phrased in more general terms, such as implementing preventive measures for their older patients during heat waves (Grewe et al. 2014).

### Responsibilities based on epistemic authority as medical scientists

We identified a broad range of responsibilities that are linked to the academic and scientific knowledge and skillset physicians possess, irrespective of whether they are treating patients or not, and on how they are publicly perceived as holders of such knowledge. In other words, we encountered a number of calls to assume responsibilities referring to the epistemic authority physicians have as medical scientists.

#### Responsibilities to identify new risks to health arising from the climate and environmental crises

Calls were prominent that assign physicians the task of identifying new health risks. Some authors phrased such responsibilities rather weakly, such as having to take environmental factors seriously despite their complexity (Wiesmüller et al. 2003) and, for example, requiring emergency physicians to maintain a high level of alertness for novel diseases in their communities due to the changing spread of vector borne diseases (Sorensen et al. 2020).

Others called upon academic medical scientists to assess environmental risks and generate evidence on the interaction between non-transmittable diseases and climate change (Avilez et al. 2016). Another author addressed dentistry, his own discipline, and emphasised the responsibility to generate robust knowledge to anticipate, mitigate, and respond to the effects of climate change on oral health (Camus Jansson 2025).

It was also common to call for specific actions, such as to ensure that health co-benefits of environmental policies are recognized and quantified (Roberts 2009), to extend the professional attention to the local community’s health, for instance by making specific reference to water pollution (ten Have 2022), and to instigate research in environmental health with special consideration of high risk groups and biological indicators of vulnerability (Velasco-Suárez 1993).

Some calls were not directed to physicians alone. In some cases this was stated implicitly, when physicians were asked to collaborate in the identification and handling of new climate-related hazards in multidisciplinary research and policy work (Lim et al. 2022).

There were also stronger positions stating the importance of involving other academic disciplines more explicitly. For example, by stating that physicians need to work with and learn from other professionals (Rapport et al. 2003), acquire a transdisciplinary perspective to advance transformation (Hübner et al. 2024), and push back the idea that all health problems are to be dealt with by professionals in healthcare delivery systems alone (Fuller 2023).

#### Responsibilities due to being perceived as a role model due to medical knowledge

As holders of special medical knowledge, it was asked from physicians to engage in good climate practices in their own behaviour as this would be well visible and likely be replicated by patients (Frank 2011) and others (Mezger et al. 2023). As role models, physicians should take responsibility as trendsetters (Risner 2020), convince others of the importance of personal behaviour changes to contribute to climate mitigation (Jacobsen et al. 2022), and lead by example (Roberts and Godlee 2007; Coverdale et al. 2022; Gozderesi et al. 2022; Fuchsig and Scholl-Bürgi 2022).

#### Responsibilities to publicly warn others of environmental concerns and engage in advocacy

A broad range of actions were requested from physicians when it came to publicly warning others about health hazards and engaging in environmental advocacy. In this context, demands were phrased in varied ways, ranging from being spelled out as positive duties, requiring them to engage in public discourse (Faergeman 2007; Kuiter et al. 2025), to negative duties, claiming that they cannot ignore the inequitable distribution of environmental burdens and its contribution to race and class inequities (Ray and Cooper 2024) and that they cannot remain silent about the threats to global health (van Gils-Schmidt and Salloch 2023).

Informing and warning the public was seen as a key area in which physicians ought to take action (Dressel et al. 2021). They were called to translate scientific findings about what is happening to the environment and the health effects into ordinary language (Chivian 2014). More specifically, physicians should use this skill to communicate about the health benefits of addressing climate change (Yang and Sarfaty 2016) and of environmental policies (Roberts 2009; Maibach et al. 2021).

Some of these calls specifically appealed to justice dimensions. Physicians were asked to actively address environmental determinants of health and to protect the natural systems that support a viable planet for future generations (Soma-Pillay, Wium, and Pillay 2022; Moloo et al. 2023) as a matter of intergenerational justice (Velasco-Suárez 1993), and seek to reduce environmental injustices, particularly triggered by structural racism (Patel et al. 2021).

Concerning the question of who physicians should address, there were appeals to inform others at all levels (Abelsohn et al. 2013). Physicians should engage in climate communication with co-workers and patients (Mezger et al. 2023) and bring environmental concerns to area-wide group action and advocacy to encourage changes to reduce or eliminate adverse health effects (Butler 1994). These demands have also been framed more strongly: physicians should educate the public and policy-makers on the health risks of the environmental crises (Leaf 1989; Atwoli et al. 2021) and have an undeniable responsibility to promote effective interventions to protect the community (Corra 2017).

A widely requested form of action was advocacy (Campos et al. 2022; McCutcheon et al. 2022; Schwartz et al. 2006; Fuller 2023; Abbasi et al. 2024; Moloo et al. 2024). Referring to the greatest threat to health we have ever faced, a commitment to contain the climate crisis was seen as part of physicians’ tasks and responsibilities (Lehmkuhl 2019). Such demands were phrased in terms of engaging in advocacy by making use of their privileged, trusted, and well-placed position (Moloo et al. 2023; Coverdale et al. 2022; Katz 2021; Crowley 2016; Rice and Rabin 2021; Guidotti et al. 1998; Macpherson and Wynia 2017; Hughes 2024). Among these demands, we find calls to advocate on behalf of those most in need to limit pollution (Worthington et al. 2017; Donohoe 2003), to generate awareness of the impact of climate change on food security (Dressel et al. 2021; Bhagratie and Iyengar 2021), and a general appeal for planetary health advocacy (Anderson et al. 2025; Stoffers and Muris 2023).

In some instances, the importance of cooperating with others in climate action was highlighted. For example, physicians ought to promote collaborative interventions in the built environment that improve population health (Zusman and Benzil 2017), e.g. natural foliage covers away from busy roads to ease physical exercise (Jacobsen et al. 2022). Other forms of cooperation outside medicine include providing public support of climate policies (Faergeman 2007; Lodge 2015; Martin et al. 2022) and considering supporting environmental organisations with their expertise and even giving preference to companies with sustainable practices (Auerbach 2008).

There was no consensus in the literature on which role physicians ought to take in climate action and how much commitment they ought to show. While some ascribed physicians a mediator role (Clar et al. 2022), others claimed they should take leadership (Lodge 2015; Solomon and LaRocque 2019) or phrased more strongly that they should not shirk a leadership role to respond to climate change (Stott and Godlee 2006) and have a direct responsibility to demonstrate substantive leadership (Jackson and Naumoff Shields 2008). Others stated that physicians have a potential “healing role” for planetary health (Moser et al. 2017). While some calls phrased appeals to take action in general terms, such as stating that they should be decisive (Haines and Ebi 2019) or to put one’s engagement with the climate crisis in one’s daily agenda (Ares Camerino et al. 2024), others considered the responsibility to engage in civil disobedience as a form of protest on top of that (Bennett et al. 2020).

Furthermore, we found that calls to work with others were stronger when there was a reference to public policy. We identified demands claiming that physicians ought to be willing to work with allies outside of the medical arena to address climate change with a health equity lens (Anderson and Walters 2022) and should work with policy-makers to address public health issues (Parker 2011; Palmeiro-Silva et al. 2020). When it came to lobbying, physicians assigned themselves major tasks as a professional group, for instance by having to lobby to ration carbon within sustainable limits (Roberts and Godlee 2007) and for stringent environmental policies (Martin et al. 2022), or making it their duty as healers to order an end of fossil fuel burning (Rice and Rabin 2021). Similarly, it was claimed that health professionals should develop a powerful, passionate, and coordinated voice to call for concrete carbon emission reductions at all levels while encouraging worldwide participation (Stott 2006).

### Responsibility based on influence on management

In this category we grouped calls where it was recognized that physicians have a significant influence over management decisions in their workplaces. It was also recognised that they have influence on the broader health sector, including supply chains and pension funds. A recurrent implicit assumption was that such power came with responsibilities to implement good environmental practices and make sure healthcare systems are resilient to climate-related disruptions.

#### Responsibilities to reduce the environmental footprint of the health sector

It was widely recognized that physicians as a group had some influence on management decisions in the institutions they worked in and that they should insist on transformation towards cutting emissions (Solomon and LaRocque 2019) and environmentally responsible behaviour (Guidotti et al. 1998) depending on their professional position.

Appeals to reduce the environmental footprint in the healthcare sector in general were very common (Katz 2021; Campos et al. 2022; Wortzel et al. 2022; Vacharathit et al. 2022; Picano 2022; Pradere et al. 2023; McCutcheon et al. 2022; Cabrera López and Cabrera Navarro 2023; Moloo et al. 2024). The duty to reduce the environmental footprint (Richie 2023) – in particular environmental toxins and greenhouse gases (Cotton and Cohen 2010), plastic waste (Saha et al. 2023), and more generally to protect the environment (Mezger et al. 2022) – was commonly understood as a requirement of the widely acknowledged principle of “primum non nocere” or “non-maleficence” in biomedical ethics. The urgency of climate action was also discussed by claiming that physicians must consider what can be done to reduce the carbon footprint *now* (Muret et al. 2019; Atwoli et al. 2021).

Frequently, calls for reducing the environmental footprint were linked to specific actions. For instance, physicians were asked to opt for environmentally friendlier alternatives when they have similar benefits (Schwartz et al. 2006), supply laboratory and clinical research to document harmful effects on the environment (Butler 1994), promote telemedicine (Lawaczeck et al. 2024), and local ambulatory care (McCutcheon et al. 2022). To respect autonomy and avoid eroding trust, it is important to engage in sensitive patient-centred communication when suggesting environmentally friendlier options, such as inhalers with less impact on the climate, and to discuss the urgency of choosing these to minimise environmental harm (Parker 2023). In terms of prescribing pharmaceuticals, physicians were asked to reconsider practices that lead to wastage (Richie et al. 2023).

On other occasions, these calls appealed to wider ecological principles, such as the responsibility to align skills and practice to sustainability (Rapport et al. 2003; Iyer et al. 2021; Macpherson and Hill 2017; Hughes 2024), participating in resource conservation efforts in the health sector (Ryan et al. 2020), protecting vulnerable non-human members (Donadoni and Ciliberti 2024), and working together to make health systems sustainable and protect planetary health (Sainsbury et al. 2019).

Implementing green solutions was another type of call. To justify it, authors appealed to multigenerational responsibility to deliver healthcare with solid efforts to mitigate environmental impact (Blau et al. 2016) and to lead by example by incorporating green solutions in offices and clinics (Abelsohn et al. 2013).

A more general appeal asked physicians to apply a climate lens to everything they do, from patient care to supply chain and facility management (Mallon et al. 2023). Similarly, there were calls to advocate for system-level changes, including healthcare financing, organization, and delivery (Resnik and Pugh 2023; Hughes 2024). Particularly in the context where physicians are employees, it was requested that they should participate in dialogues about the carbon footprint at their institutions or even build “green teams” to advance the implementation of energy conservation measures and recycling (Koch and Pecher 2020; Guidotti et al. 1998).

Complementing these general calls, we identified several health disciplines issuing specific calls for action. For example, surgeons and oncologists must take responsibility for reducing emissions of gasses with very high global warming potential (Lichter et al. 2023; Koch and Pecher 2020) and rethink daily surgical practices, including to reduce the amount of unused items in surgical sets (Vanni et al. 2023; Bellaire 2025). A radiologist inspired by the Choose Wisely campaign requested their colleagues to factor in the environmental footprint in their decision-making for medical imaging (Picano 2022). Paediatricians were asked to work with children and their parents to reduce the footprint in healthcare and beyond (Gozderesi et al. 2022). Implicit in these calls was that each health discipline has good knowledge and opportunities to align the practices in their domain with sustainability goals.

There were also some attempts to align financial and administrative tools with environmental protection goals and their professional ethos. For instance, physicians were asked to promote sustainable investment strategies for the health sector’s pension funds (Schulz et al. 2019). There were also calls for fossil fuel divestments (Atwoli et al. 2021; Schneider et al. 2022) by referring to the ability of today’s children to live healthy lives now and in the future (Jee et al. 2023). At the management level, there were requests to include sustainability policies in quality assurance (Dyer et al. 2023) and quality improvement routines (Blau et al. 2016).

#### Responsibilities to prepare the healthcare sector for environmental and climatic disruptions

Climate change is already a major stress factor for the healthcare sector and calls stated that physicians will have to make adaptations. For this, healthcare systems will need to be prepared for major natural calamities and physicians will need to be ready to work and make difficult decisions in austere environments (Pingree et al. 2020).

At the individual level, physicians need to remain hopeful despite the obstacles created by political denial and delaying tactics so that they can work towards fulfilling their obligation to protect public health from climate-related risks (Jameton 2017). Extreme events triggered by climate change will require preparations to respond in emergency medicine (Salas 2020; Sorensen et al. 2020), and improve climate disaster preparedness in general (Fuller 2023). Thus, physicians will need to prepare the health system for having to respond to massive demands during climate-related disruptions, such as heat waves, wildfires, floods and hurricanes (Jacobsen et al. 2022), and deal with the special immediate and long-term health hazards of wildfires due to drier climate conditions (Rossiello and Szema 2019). In addition, they need to recognize that certain medicines may conflict with heat regulation during heat waves (Sorensen et al. 2020).

Bioethics itself also needs to be adapted to the new environmental challenges so it can effectively guide healthcare practice in the Anthropocene (Anderson et al. 2025). Physicians were also asked to help “reimagine” bioethics to deal with the contemporary climatic and environmental challenges (Diller and Williamson 2023) and become actively involved in ethical debates on balancing environmental responsible healthcare with patient-centred clinical practices (Jameton and Pierce 2001).

Two authors pointed out that this would require revisiting early bioethics to recognise that anthropocentric and ecocentric interests are intertwined (Velasco-Suárez 1993) and to adopt a view of the moral world that understands a person as both an individual and as a member of the ecological community (Wardrope 2020). In contrast, others proposed applying newer ethical approaches to medical reasoning. For instance, by arguing that planetary health principles need to be rooted in the professional ethos (Wabnitz et al. 2020), or by linking ecologically conscious clinical virtue with an ethics of care, relational autonomy and climate justice to ground deep commitments to the environment that clinical medicine must meet (Hantel et al. 2025). Furthermore, physicians ought to pay attention to cultural differences in relation to the (home) environment and its role for patients’ well-being (Coward and Sidhu 2000; Braun, Mokuau, and Tsark 1997).

### Responsibilities as citizens

We could also identify a few calls for action that were more broadly directed to physicians as citizens or ethical agents who are free to adapt their choices, irrespective of their medical skills and knowledge. For instance, for physicians like any other professionals who frequently travel for further training, there are appeals to be selective when attending conferences and to consider online options (Storz 2019). Similarly, as is the case with other people working in large institutions, those working in medical institutions – including physicians and bioethicists – should also help in reducing the impact of their workplace on the environment, particularly considering that hospitals have a huge carbon footprint and thereby harm public health (Churchill et al. 2024).

It was also claimed that improving the environment and mitigating climate change was everyone’s responsibility, therefore physicians had to do their part (Butler 1994; Vanni et al. 2023), or framed negatively, physicians were not exempt from the obligation to reduce carbon emissions (Vacharathit et al. 2022). All professions – without exceptions – need to ensure that skills and practices result in sustainable systems (Rapport et al. 2003).

Another position argued that special skills and a privileged position increase obligations to reciprocate to society and that greater ability to prevent harm leads to greater responsibilities to do so – a call specially directed to citizens that are better off, such as physicians (Shrader-Frechette 2012).

## Discussion

As we have identified in our review, the large number of ethical responsibilities in relation to climate and environmental changes can be related to four major roles of physicians as: (a) medical practitioners, (b) medical scientists, (c) facility (co-)managers and (d) citizens. However, calls to assume special responsibilities were rarely explicitly grounded in further or deeper ethical reasoning. That is, most sources listed responsibilities without further explanation or deliberating reasons. We proceed by briefly discussing a few direct – albeit mostly implicit – attempts made to provide ethical grounding for these responsibilities in selected publications. We then go on to discuss the many spheres physicians are being called to act in, and lastly argue for more ethical inquiries and research into whether, and which, responsibilities physicians ought to assume in times of climatic and environmental change.

### Ethical grounding of responsibilities

In general, when asking others to assume certain responsibilities – particularly when these limit freedom and regulate behaviour – requests for action become more convincing when they are grounded in ethical reasoning. However, most of the calls we reviewed did not explicitly attempt to provide grounds on why physicians in particular ought to assume responsibilities beyond their current realm of professional activity. Nonetheless, there are a few references to foundational ethical concepts worthy of attention (see Table 2).

**Table 2:** Summary of frequently used responsibility-guiding principles

Responsibility-guiding principles	Translations of these principles into practice	Links to identified responsibilities	Sample references
Respect autonomy	Patients need to be provided with information on the environmental footprint of their intervention so that they can make autonomous decisions	Responsibility to inform patients	(Richie 2023; Cohen et al. 2024)
Avoid harm	The imperative of not doing harm needs to be extended to avoid that the environmental footprint of the health sector has a negative effect on the health of others	Responsibilities to reduce the environmental footprint of the healthcare sector	(Mezger et al. 2022; Cotton and Cohen 2010; Saha et al. 2023)
Address intergenerational justice	The environment needs to be protected so that future generations can have the opportunity to live healthy lives	Responsibilities to publicly warn others of environmental concerns and engage in advocacy Responsibilities to prepare the healthcare sector for environmental and climatic disruptions	(Soma-Pillay, Wium, and Pillay 2022; Moloo et al. 2023; Velasco-Suárez 1993; Kenner et al. 2023)
‘Can’ implies ‘ought to’	Physicians have special skills, are trusted and have special knowledge due to their position in society which makes them particularly well suited for advancing environmental protection. These assets come with responsibilities	Responsibilities to publicly warn others of environmental concerns and engage in advocacy Responsibilities as citizens	(Shrader-Frechette 2012; Rice and Rabin 2021)
Doing one’s share	Everyone needs to do their part in protecting the environment and halting climate change, including the health sector	Responsibilities to reduce the environmental footprint of the healthcare sector Responsibilities as citizens	(Butler 1994; Vanni et al. 2023; Vacharathit et al. 2022)

The influence of Beauchamp and Childress’ Principles of Biomedical Ethics (2019) in medical education was clearly visible, as authors frequently made reference to these four principles. For instance, the importance of providing information on the environmental footprint of medical interventions or devices and climate-related health hazards – especially in cases where this is valued by the patient – was often defended as a measure to improve patient autonomy (Richie 2023). The application of other principles to deal with the climatic and environmental crises required some reinterpretation by the reviewing authors. For example, we found sources that tended to interpret the principle of non-maleficence – or *primum non nocere* – quite broadly, as not only the duty to avoid direct harm, but also as reducing the negative side-effects of the activities within the healthcare sector (Moloo et al. 2023; Wabnitz et al. 2020).

Similarly, some calls can be loosely interpreted as being based on ramifications of the principle of justice, even though these were rarely extensively discussed in terms of (environmental) justice or equity (Maibach et al. 2021; Atwoli et al. 2021). Here we can identify positive duties, such as doing one’s share in addressing the climate and environmental crises, and occasionally, although mostly implicitly, negative duties to avoid free-riding in efforts to improve public health. While there is a long tradition among physicians to address issues of social justice, it was rare to find calls that saw it as part of medical responsibilities to address and rectify environmental *injustices* (Ray and Cooper 2024), except for when these jeopardised the possibilities of future generations to live healthy lives (Donohoe 2003; Kenner et al. 2023).

Some strong calls were backed by arguing that physicians had unique skills and knowledge which made it difficult or impossible for others to replace them. For example, in their role as practitioners, family physicians have specific knowledge on their patients’ needs and vulnerabilities (Lodge 2015; Grewe et al. 2014; Cabrera López and Cabrera Navarro 2023). Another claim was that professional privileges required them to give back to society by assuming special responsibilities (Shrader-Frechette 2012).

On other occasions, it was argued that the special responsibilities were in line with traditional responsibilities both practitioners and medical scientists had assumed in the past, such as raising awareness about the catastrophic dangers of nuclear war (Schwartz et al. 2006). Moreover, it was claimed that physicians already pursue such actions quite effectively, e.g. because they are trusted and have the capacity to explain scientific matters in plain language (Gusmano 2019). Due to the urgency of the climatic and environmental crises, it was often claimed that physicians – irrespectively of whether they communicate only to patients or the wider public – ought to assume responsibility because they are likelier to succeed in convincing others about taking action (Sarfaty and Abouzaid 2015; Shrader-Frechette 2012).

### Spheres of action

While much of medical ethics concentrates on the patient-physician encounter, particularly in the clinical setting, calls to assume responsibilities in relation to environmental changes went beyond the individual patient-physician interaction, requiring physicians to act at various levels, as individuals and as a professional collective.

Some responsibilities, such as informing patients about the environmental footprint of medical interventions and the health co-benefits of plant-based diets and active transportation, can be fulfilled within the traditional patient-physician encounter.

For other responsibilities, physicians would have to adapt their management practices or get involved in management decisions at the institutional level to promote sustainable practices.

**Fig. 3:**
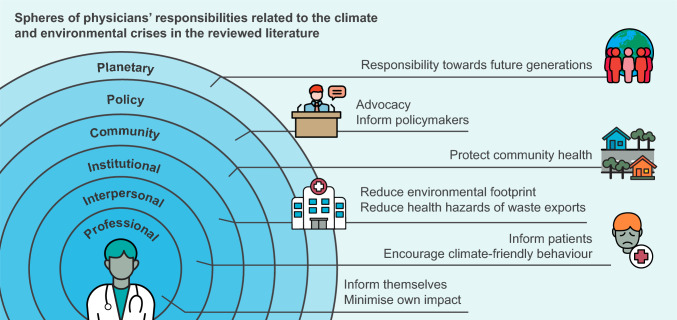
Identified spheres of physicians’ responsibilities

Furthermore, physicians would have to engage in public communication at different levels, from the local to the global, to influence policy-makers and even be in dialogue with colleagues around the world (see Fig. 3).

In sum, according to our findings, physicians should be taking action on all levels, from the individual, to the organisational and also as an organised professional collective on a political level, as has also been done in the past in calls to abolish nuclear weapons, contribute to poverty relief and reduce alcohol and tobacco consumption (Gusmano 2019; Schwartz et al. 2006).

### Need for further research and debate on revising medical responsibilities

The reviewed literature clearly indicates that more research and debate is necessary to discuss, ground and justify physician responsibilities for the environment and climate.

In general, there is a strong need for future bioethics and environmental ethics research to assess and ground physicians’ ethical responsibilities towards the environment and climate. In our view, this task would require diversifying bioethics approaches, e.g. by building on criticism of principlism (Wardrope 2025), particularly concerning the still underexamined principle of justice (Ray and Cooper 2024; Timmermann et al. 2024), and by expanding on the grounds of feminist (Sherwin 2008) and decolonial bioethics (Sachdev et al. 2025), including from Indigenous perspectives (Redvers et al. 2022), and on existing literature from environmental and climate ethics (Dwyer 2009; Gardiner and Tubig 2023). These efforts must be further supported by a reconceptualization of the patient as an ecological being – one whose health is shaped by the interactions of microbial communities (Beever and Morar 2016a, b) and by the dependencies of human health on local ecosystems and planetary health (Traidl-Hoffmann et al. 2021). In our view, building on these areas provides fertile grounds for discussing and grounding physician responsibilities further, and for delivering valuable material for future collaborative research for example with health, environmental and social sciences. Such a collaboration could help to assess how to prioritise and balance physician responsibilities, to examine whether and how different specialties or contexts might lead to different responsibilities and how to include the responsibilities into professional identity formation (Wild 2026). Discussing physician responsibilities also opens the field towards fundamental questions of anthropocentrism (Wardrope 2020). How would such responsibilities change if a holistic, multispecies approach was underlying (Deane-Drummond 2019)?

Future research could also expand on examining best practice examples and challenges, but also barriers in implementing responsibilities. Even though our search strategy and review did not focus specifically on identifying dissenting voices, there were a few positions against taking on environmental responsibilities worthy to point out, that could and should be explored in further health, environmental, social science and ethics research. For example, some argue that physicians ought to concern themselves primarily with the health of their patients and addressing sustainability concerns could put them in a conflict of interest, which may erode trust in the health system and push physicians to disregard the patient’s best interest (Parsa-Parsi et al. 2024; Wiesing 2022). Furthermore, ill-informed attempts to reduce emissions might have the opposite effect, particularly when physicians make recommendations that do not weigh in factors such as commuting to pharmacies and practices (Armitage 2023). Another concern was to question whether physicians fighting for environmental protection were acting beyond their competency, and that this might undermine trust in them and the health system (Karches et al. 2021). Others were more cautious in making such claims. For instance, it was discussed that one ought not to overestimate the current level of trust physicians have and the likelihood that they can maintain this level of trust if they fail to act on social challenges. On the contrary, social activism may even restore trust in the profession (McCally 2002).

We could also observe a broader concern on whether focussing too much on physician’s roles and responsibilities may deviate attention from the magnitude and wickedness of environmental and climate crises. As wicked problems, they require collective action, including top-down and bottom-up solutions, and effective leadership. Attributing responsibilities to physicians for environmental issues that demand systemic change and governmental action may frustrate and impose undue pressure on already overburdened professionals (Yassaie and Brooks 2025; Lignou and Hart 2024). Furthermore, some authors argued that responsibilities are dependent on existing infrastructure, institutional policies and healthcare insurers (Komparic et al. 2025; Richie 2025). Environmental factors are becoming a too strong determinant of health to remain within the responsibility of physicians and even health authorities, requiring wider social and political responses at all levels (Rodríguez Bertheau et al. 2011).

Further research could systematically discuss such concerns and criticisms, and thereby develop further the grounding and justification for physicians’ ethical responsibilities, and their potential limitations.

We are indeed beginning to see ethical work providing a more nuanced discussion of solid grounds for these ethical responsibilities (Tarver and Macpherson 2025; van Gils-Schmidt and Salloch 2023; Wild 2026). A first overview of the task ahead is provided by a recent literature review that has analysed the different reasons presented in favour or against physicians including aspects of environmental sustainability in their clinical decisions (Kuiter et al. 2025).

We can also see first attempts to provide concrete guidance. For example, Macpherson and Wynia (2017) identified seven criteria which allow individual physicians to determine the extent of their environment-related responsibilities, such as proximity to the problem and costs of taking action, among others.

The position that physicians ought to consider only a patient’s best interests has been criticised as being no more than a “lofty ideal”, as physicians have traditionally acted as gatekeepers of valuable resources, such as expensive drugs, diagnostics and antibiotics, where they must balance their commitments to patients with their obligations to the good of society (Doernberg & Truog, 2023). Looking out for the best interest of patients shall not serve as an excuse to fully ignore the environmental footprint of healthcare, as the patient-physician relationship does not exist in a social vacuum unrelated to other ethical concerns (Bhopal and Bærøe 2023). To facilitate greener clinical decision-making, it has been suggested that institutional guidelines place the default on “greener options” and provide a general clause left to the individual physicians’ discretion to deviate from sustainability goals (Herlitz et al. 2023). Drafting and communicating guidelines to align clinical practice with environmental protection can also reduce the pressure on physicians. Here, too, more bioethics and environmental ethics research is needed to develop guidance for professionals, that is theoretically sound, participatory in its development, and that fits to real world contexts, needs and demands.

## Limitations of this review

In this review, we could only include sources published in English, German and Spanish. We acknowledge that despite our efforts to diversify sources, we still fear that we may not have captured sufficiently diverse ways of framing ethical responsibilities around the world. For instance, some authors opted to assign responsibilities to health systems (Hancock 2023) or issued broader calls to rethink the role of the environment in medical ethics (Cummins 2018) – thus falling outside the scope of this review. It is likely that we could not match this diversity with our selection of keywords for our searches, and thereby failed to identify many non-Western contributions, including when cultural or political norms might lead to hesitancy in formulating calls for action explicitly. To reduce the amount of material for analysis and discussion, we were obliged to exclude important topics, such as the role of physicians in reducing the health risks of nuclear disasters, indoor pollution and zoonotic diseases transmission. Further work needs to assess physician responsibilities in relation to these issues.

Furthermore, we recognise that reviews come with biases in the selection, identification and grouping of source materials and their review. We tried to reduce the bias as far as possible through extensive group discussions of the results. Lastly, we want to emphasise that reviews provide an overview of the literature at a particular point in time.

## Conclusion

On the basis of an extensive literature review, we mapped the different ethical responsibilities physicians are being asked to assume in view of the environmental and climatic crises. We see that urging physicians to take on responsibilities for the environment are becoming widely spread and starting to be anchored in professional guidelines. The large number and diversity of calls we identified show that more work is needed to assess which responsibilities physicians are being asked to assume are ethically justified, when and how this should be done, and what impact fulfilling – or ignoring – these responsibilities might have on the medical profession, the environment and society at large.

This review provides ground for future ethical analysis, to which further research and debate ought to provide answers: How to include ethical responsibilities towards environmental and climate crises into medical and continuous professional education? How to reach the necessary diversity in voices and perspectives when defining responsibilities? How to address and balance potential tensions between responsibilities, including those that are likelier to be more urgent in the future? And who should be bearing those responsibilities alongside physicians, why and how? We would also welcome future multidisciplinary research that examines the risks of underinformed physician advice on environmental matters and assesses the psychological stress of further expanding the scope of medical responsibilities. As the environmental and climatic crises and their health impacts intensify, we urgently need answers to these questions.
